# A thing about karate in physical culture

**DOI:** 10.3389/fspor.2024.1430186

**Published:** 2024-07-26

**Authors:** Paweł Adam Piepiora, Petra Čaplová, Wojciech Jan Cynarski

**Affiliations:** ^1^ Faculty of Physical Education and Sports, Wroclaw University of Health and Sport Sciences, Wrocław, Poland; ^2^ Faculty of Sciences, Humanities and Education, Technical University of Liberec, Liberec, Czechia; ^3^ Institute of Physical Culture Studies, College of Medical Sciences, University of Rzeszow, Rzeszów, Poland

**Keywords:** karate culture, martial art, movement science, physical activity, qualitative study

## Abstract

This paper deals with karate activity in the areas of physical culture in the broad sense. It is a concise and clear approach to the topic, which aimed to conceptualise the inclusion of karate in areas of physical culture as a contemporarily attractive physical activity. Karate was described here as a combat sport, martial art, and self-defence system in the following areas of physical culture: physical education, physiotherapy, recreation, sports, and tourism. Reference here was made to physical activity focused on bunkai (circuit interpretation exercises), kata (circuit exercises), kihon (technical exercises), and kumite (combat exercises). It has been determined that practitioners’ involvement in karate culture affects their level of sense of coherence. In addition, the conditioning for using karate in physical culture to the maintenance of health was mentioned. Here, reference was made to the perceptual perspective of the bodily and mental practice of karate for health. It has been established that this can be effective by adopting only the objectives of karate as martial art. Based on the above, it was concluded that practising karate is present in all areas of physical culture as combat sport, martial art, and self-defence system, but practising karate only as martial art provides opportunities for maintenance of health.

## Introduction

Nowadays, karate (empty hands) is one of the most popular fighting methods, as it is trained in all countries of the globe in many style variations (1). But this phenomenon emerged gradually. First, karate became known as a method of self-defence without the use of weapons. It had been forming for centuries in Okinawa because of the fusion of indigenous fighting techniques with Chinese varieties of confrontation (2). It is worth noting that at the time, the use of karate techniques in self-defence was regarded as a fight to the death (3). At the beginning of the 20th century, thanks to the efforts of Gichin Funakoshi and courtesy of Jigoro Kano, karate began to spread in Japan. In 1936, karate was recognised as a martial art belonging to the budo (Japanese fighting methods) (4). To emphasise the psychophysical nature of this martial art, the name karate-do (the way of the empty hands) has come to be used (5). After the Second World War, karate masters sought to make their martial art sporty, and many associations were formed that adopted different philosophies of sporting confrontation (6). The fact is that the interest in stylistic sports competition in karate contributed significantly to the global popularity of karate. The rest was completed by the mass media and the promotion of karate in films and video games (7). Finally, after several decades, the International Olympic Committee recognised karate as an Olympic discipline in 2016 (8).

Accordingly, contemporary karate is a combat sport, martial art, and self-defence system, as the practice of this fighting method is different depending on the training goals adopted (9). Thus, training karate as a combat sport involves preparing for and participating in seasonal competition, where the best sporting result is desired. In turn, training karate as martial art involves self-improvement through psycho-physical training, the levels of which are reflected in the hierarchy of student kyu and master dan degrees. In contrast, training karate as a self-defence system is based on improvement in proficiency in non-sport combat. With the above issues in mind, karate has been found to follow budo (10), theory of combat sports (11), theory of combatives (12), and general theory of fighting arts (13). It is worth noting that karate masters prefer teaching which is relevant to their fighting philosophy. Therefore, different karate associations adopt different trends that fit into one or more of the above-mentioned theories. This means that the adopted training goals set the direction of karate practice entirely oriented towards combat sport or towards martial art or towards the self-defence system, or towards a specific one overarching goal (e.g., combat sport) and two subordinate ones (e.g., martial art and self-defence system).

Consequently, the modern understanding of karate is not dependent on styles, but on training objectives. And this is fully in line with the currents of traditional, sport and Olympic karate. Therefore, a holistic understanding of karate indicates the great versatility of this fighting method (14). But already a partial understanding of karate (for example: reasoning only from the perspective of style varieties: Goju-ryu, Kyokushin, Shotokan, etc.) indicates the weakness of this fighting method (for example: judo has more advantages than a given karate style) (15). Therefore, the assumption of a holistic understanding of karate as a combat sport, martial art and self-defence system was made, as this identifies the physical and mental aspects of this fighting method (16). Therefore, the aim of this article was to conceptualise the inclusion of karate in the areas of physical culture as a contemporarily attractive physical activity.

## Karate in physical culture

Considering the outlined directions of karate practice, the significant thing is that they are fully part of physical culture. That is, they fall within the general activities in accordance with the principles and norms of acceptable behaviour, which are aimed at the proper psychophysical development of society, in which health in the broadest sense is the goal (17). These behaviours are part of a general culture in caring for physical development, physical fitness, health, beauty, physical perfection, and human expression, following patterns accepted in a given community, as well as the results of these behaviours (18). The emphasis here is on the harmonious development of a versatile, mature personality and health in the physical, mental, and relational spheres (19).

Physical culture can be experienced in five areas: physical education, physiotherapy, recreation, sport, and tourism (17). Physical education prepares for lifelong care of one's body through physical activity, taking care of one's physical fitness and health. It is the preparation of children and young people for a physically active adulthood (20). Physiotherapy, on the other hand, is the use of the natural movement of the human body for rehabilitation purposes using physical factors found in nature and the environment. It is the maintenance, development, and restoration of a person's impaired motor abilities to improve function throughout life (21). The third area, recreation, is physical activity undertaken outside of work, social, domestic, and academic responsibilities. It is invariably used for leisure, rest, and entertainment to relieve nervous tension and prevent civilisational diseases (22). In turn, sport is competition in various forms of physical activity, according to specific rules, aimed at achieving sporting results at the following levels: amateur (competition for entertainment); competitive (competition for the best result); professional (competition for the best result and earnings). It is a structured physical activity in macro-cycles, mesocycles and micro-cycles of training (23). The last area, tourism, is all phenomena of temporary and voluntary travel that are related to changes in the environment and rhythm of life. They refer to personal contact with the natural, cultural, and social resources of the area visited. Physical activity here is oriented towards cognition, experience, leisure, and entertainment (24).

The bodily practice of karate refers to physical training focused on bunkai (circuit interpretation exercises), kata (circuit exercises), kihon (technical exercises), and kumite (combat exercises). In the various areas of physical culture, the training emphasis is distributed differently.

Karate in physical education is implemented through exercises: technical, circuits and their interpretation, and fighting. It is important that for the bodily safety of the education of children and adolescents, fighting exercises are elementary (25). In physiotherapy, on the other hand, technical exercises are emphasised in motor rehabilitation. Only at the final stage of movement treatment are circuit exercises recommended to verify the biomechanics of the patient's musculoskeletal system (26). In the following areas of physical culture: recreation, sport, and tourism, all groups of karate exercises are present. In recreation, the main factor is the entertainment of karate by practising circuits or conventional fights (27). In sport, the emphasis relates to the specialisation of the athlete: kata or kumite, i.e., targeting refers primarily to the practice of circuits or fights according to the rules of sports competition of a specific karate organisation (28). In tourism, the activity consists of trips to destinations where the highlight is to connect with karate culture to pursue practical studies or research (29).

Therefore, it was concluded that the directions of karate practice (combat sport, martial art, self-defence system) in physical culture (physical education, physiotherapy, recreation, sport, tourism) introduce the culture of karate. This is manifested in the gradually increasing sense of coherence of karate adepts as they acquire and improve their fighting skills (30). The sense of coherence manifests itself through the comprehensibility, manageability, and meaningfulness of engaging in and creating one's own life (31). Comprehensibility manifests itself through an understanding of the purposefulness of practising karate in a given area of physical culture. Manageability determines the extent to which a karate practitioner perceives the resources available to him/her as sufficient to overcome any difficulties in life. Meaningfulness determines the extent to which a karate practitioner feels that his or her life has meaning, that it is worth the effort and commitment to meet the demands of life. There is a visible interaction here between body and mind−between physical and mental training.

In physical training, practising karate involves continuous development of physical fitness through the acquisition and improvement of fighting skills. The emphasis here is on motor skills and technical and tactical skills (32). In mental training, on the other hand, practising karate involves continuous development of mental potential through the acquisition and improvement of psychological skills. The emphasis here is on: controlling aggressiveness, creating health-promoting attitudes, imagination, maintaining attention, mastering emotions, mental toughness, motivation, self-confidence, relaxation, triggering prowess, and visualisation (33). Hence, the unity of body, spirit and mind is maintained in karate culture through physical and mental exercises. It is important to distinguish between these variables which are positively influencing health. But in principle—an individual's involvement in a karate activity in a given area of physical culture will depend on their interests and health capabilities.

Therefore, when referring to the maintenance of health through the practice of karate, the possibility of using this fighting method as an excellent physical activity in all areas of physical culture has been noted: physical education, physiotherapy, recreation, sport, and tourism. It is important that the goals of practising karate in physical culture must be oriented towards martial art. Traditional values and the internalisation of karate principles by the karate culture come to the fore (14). The training emphasis relates to an emotional approach to karate, respect for authority and maintaining mental hygiene (34). Emphasis is placed on the principle of non-violence. Practitioners learn through combat training how to avoid fighting (35). Through practising karate, one strives for psycho-physical improvement and self-realisation (36).

Also important are the philosophical, ethical and moral influences maintained by the rank hierarchy (37). These relate to traditional budo values and are embodied in etiquettes of conduct in and out of the dojo (38). This is noticeable in the relationship between teacher and student, between senior and junior ranks, and between people of equal degrees. Here, the physical and mental development experienced in practicing karate relates primarily to overcoming one's own weaknesses and limitations (39). Thus, respect for moral norms is combined with the formation of practitioners’ self-control and emotional zone (40). The idea is that knowing your own weaknesses and overcoming them is the surest way to elevate all your skills. Therefore, the goals of karate as a martial art, understood in this way, minimize the risk of injury and trauma among practitioners and are aimed at maintaining health (41). This is not so noticeable in the goals of karate as a combat sport and self-defense system, which have inherent in the physical activity the likelihood of injuries and trauma among practitioners (42). This is related to direct confrontations between competing opponents in sporting bouts (43) or in non-sporting bouts (44).

## Discussion

To practise karate in physical culture, training should be martial art-oriented and tailored to the level of the practitioners. Therefore, in physical education, recreation and tourism, groups of exercisers should be determined by age and training level. In physiotherapy, on the other hand, karate activities must be adapted to the individual abilities of those being rehabilitated. This is determined more by movement dysfunctions and motor abilities than actual fighting skills. On the other hand, preparation for sports competitions (here, seasonal rivalry will also have the characteristics of combat sport) should be conducted in different age groups, depending on the level of the practitioners and their specialization in kata or in kumite. But only competition in kata minimizes the occurrence of injuries and trauma and excludes the tensions of direct confrontation.

The role of a competent coach or teacher who can skilfully shape the psychophysical development of practitioners is essential here (45). Coaching, on the one hand, and interest and involvement in karate culture, on the other, create a specific mechanism for health (46). It manifests itself in a passion for karate (47). It is therefore legitimate to use physical and mental karate exercises (48). And in this sense, karate as a combat sport, martial art and self-defence system occurs in all areas of physical culture, but only as martial art can it be used to health.

However, inappropriate practice of karate may bring negative effects. Apart from injuries and trauma (49), poor teaching can result in the development of disturbed personalities (50), negative attitudes (51) and movement habits (52) among practitioners. Therefore, it is worth mentioning that a contemporary threat to karate culture is the continued escalation of mixed martial arts, where attempts are made to equate brawling with martial arts (53).

The assumptions of mixed martial arts confrontation have nothing to do with the philosophy of martial arts confrontation. Furthermore, it is noted that the escalation of mixed martial arts is increasingly being camouflaged in the media under the martial arts banner to gain public approval for the formal status of the sport. It is therefore legitimate that this phenomenon of the pathology of mixed martial arts is consistently referred to as neogladiatorism (54). Only such an approach guarantees the social identification of what is health-promoting and what is unhealthy. And in this sense, neogladiatorial violent techniques of fighting and overthrowing cannot be pro-health, because they epitomise the massacre of people confronting each other and the motives of neogladiatorial confrontations are reduced to brawling.

A final issue is the utility of karate. Proponents of karate emphasise the educational and traditional values of this fighting method, which positively translate into the social functioning of karatekas. Opponents of karate, on the other hand, criticise this fighting method as questionable and ineffective *in situ*ations of real danger to life or health. It should be categorically stated here that there is no fighting method that gives one hundred per cent chance of an effective defence. The fact that one trains in karate or another fighting method only increases the likelihood of coming out of a dangerous situation unscathed. Therefore, the effectiveness of these actions will be conditioned differently by personality among all those training different fighting methods.

## Limitations of the perspective

This perspective is the authors’ subjective opinion on karate in physical culture. It is encouraging that this form of physical activity is becoming increasingly popular. It is noteworthy that research on karate in the field of sport is being systematically disseminated. But research on karate in the areas of physical education, physiotherapy, recreation and tourism is still scarce. Nevertheless, the insights gathered from the analysed research results present a completely new perspective that contributes to the discourse on this topic. Therefore, an important contribution of the authors is the innovative inscription of karate in all areas of physical culture, considering contemporary combat theories. In addition, the goals of karate as a martial art are pointed out, which overlap with the goals of physical culture in the broad sense of health through physical activity. It should be emphasised that this perspective is a strong theoretical construct and considers contemporary approaches to karate—in its entirety—in the traditional, sport and Olympic strands in the semi contact, knockdown, full contact and mix fighting systems. Considering karate in terms of style or organisational divisions weakens the relevance of this fighting method. In such perspectives karate will always show weaknesses and succumb to other fighting methods.

## Directions for further action

So far, no empirical studies have been published on the holistic treatment of karate in physical culture. This article is the first to indicate this line of research. Therefore, this work is a cognitive novelty for further experimental research. It is recommended to conduct and disseminate the results of karate research from all areas of physical culture. It is also important to systematically implement this perspective of karate for social utility in physical culture.

## Conclusions

Karate fully fits into physical culture as a combat sport, martial art, and self-defence system. Moreover, karate as a martial art is the equivalent to physical culture objectives aimed at maintaining health through physical activity.

## Data Availability

The original contributions presented in the study are included in the article/Supplementary Material, further inquiries can be directed to the corresponding author.
